# Differences in Relaxation and Imagery among NCAA Division I Sport Types

**DOI:** 10.3390/sports11110224

**Published:** 2023-11-13

**Authors:** Elizabeth Warfield, Philip Esposito, Robyn Braun-Trocchio

**Affiliations:** Department of Kinesiology, Texas Christian University, Fort Worth, TX 76129, USA; e.warfield@tcu.edu (E.W.); p.esposito@tcu.edu (P.E.)

**Keywords:** relaxation, imagery, psychological skills, collegiate athletes, sport types

## Abstract

Athletes use psychological skills such as imagery and relaxation to decrease stress, cope with competitive anxiety, and achieve an optimal state of arousal. There is conflicting literature on how team and individual sport athletes use these skills, with some saying that individual sport athletes have better capabilities and others saying team sport athletes use imagery more frequently. The current study analyzed sport type differences in the use of relaxation and performance imagery among NCAA Division I (DI) athletes. This study included 117 NCAA DI athletes, including team sport (*n =* 72) and individual sport (*n* = 45). Participants completed a modified version of The Deliberate Relaxation for Sport Survey through Qualtrics. Results indicated there is a statistically significant difference in the type of relaxation technique used based on the individual’s sport type. Team sport athletes used muscle relaxation (*p* = 0.034), eastern relaxation (*p* = 0.014), and stretching (*p* = 0.020) more frequently than individual sport athletes. Additionally, individual sport athletes used performance imagery more often for mental focus than team sport athletes (*p* = 0.012). There were no differences between sport types in the level of deliberate practice of relaxation and performance imagery. Athletes used a variety of methods to learn both imagery and relaxation skills and have been using these for an average of four years. The majority of participants (*n* = 67) did not work with a sport psychology professional, but their school has one. This study supports the use of individualized intervention programs to help athletes use relaxation and imagery in the most effective ways for their performances.

## 1. Introduction

High-level athletes experience stress and anxiety associated with the pressures of their competition. To combat this, athletes use psychological skills such as imagery and relaxation techniques to decrease this stress to achieve an optimal state of arousal and focus [1]. Performance imagery is where an athlete combines multiple senses to create vivid and controlled mental imagery of themselves performing a skill [2]. This allows the individual to build muscle memory and plan an event before it happens by mentally imagining a scene or skill [2]. This decreases state anxiety by letting athletes know their bodies can perform the activity even before they begin the movement. Relaxation helps to calm individuals, which leads to an increase in confidence in themselves by allowing their body and mind to relax and focus on the correct cues [3]. The relaxation techniques most used in athletic settings and the ones focused on in this study are muscle relaxation (tensing muscles and then relaxing muscles one by one in the body), autogenic relaxation (the individual feels patterns of heaviness and warmth in each part of the body), deep breathing (paying attention to inhaling and exhaling), eastern relaxation (activities such as yoga and tai chi where limbs are put into specific postures to increase awareness over mind and body), meditation (attempts to get beyond thinking and into a deep state of relaxation or awareness), and imagery (focusing on various images in your mind that evoke feelings of ease and release stress) [4]. Imagery can also be used to increase their performance level, ffocus, confidence, motivation, arousal regulation, relaxation, and improve skill development [5,6]. Many sport psychology professionals (SPPs) encourage the PETTLEP (Physical, Environment, Task, Timing, Learning, Emotion, and Perspective) model of imagery [7] because it incorporates such specific aspects of the environment that create a strong parallel between mental and physical practice. 

Athletes typically deploy psychological skills right before or during a competition to try and combat any adverse situations they may be facing. Doing so is not an effective way to use these mental skills, though, as they require repeated practice to master [8]. The more athletes use psychological skills prior to competition, the more likely they are to see themselves as successful athletes [8]. Individuals with a strong background in psychological skills can maintain the intensity of their anxiety response before a competition with the use of goal setting, imagery, self-talk, and relaxation [9]. Athletes often use a combination of deep breathing, muscle relaxation, and imagery to prepare for an event and promote recovery after a performance [10].

Only a few studies have investigated how the use of psychological skills differs between individual and team sport athletes. There is also a gap in investigating relaxation skills, as most studies focus on imagery skills. Research has focused, in a broad sense, on how athletes use psychological skills in performances, but there is limited examination of how athletes are specifically using them, how they learned these techniques, and how these skills help their performances [11,12,13,14]. Past literature studies have found that imagery was effective for both individual and team sports, but individual sport athletes have better imagery capabilities [3]. In contrast, team sport athletes in certain sports have been found to use imagery more frequently [2,15,16,17]. Researchers found that elite athletes use deep breathing, imagery, and muscle relaxation to cope with competitive anxiety. Additionally, this study revealed that elite athletes use relaxation and imagery to promote recovery while stretching after a performance [10]. However, there is limited research on how elite athletes use relaxation and imagery before or during their performances. Research has shown that elite and collegiate athletes tend to use these techniques more frequently and significantly benefit from them more than a novice [11].

Even for an elite athlete, to use relaxation and imagery skills effectively, athletes need to use them frequently and with deliberation. Deliberate practice is structured, purposeful practice solely for enhancing performance and considers relevance, effort, concentration, and enjoyment—or the lack thereof [18]. Conflicting literature studies have found deliberate practice for performance enhancement to be enjoyable [4,19]. Deliberate practice has been shown to be the difference between novice and expert athletes. Expert athletes have completed a life-long period of deliberate practice specifically to improve their performance [18]. If athletes really do enjoy deliberate practice, they may find it easier to implement in both practice and competition, which would have more significant and impactful results on performance. Findings from this study on how to implement psychological skills based on different sport types and increase the deliberate practice of these skills could lead to more effective interventions. 

To extend and develop upon the existing literature, the overall purpose of the present study was to gain knowledge about the relevance of relaxation and performance imagery skills to athletic performance as well as the extent of use, types, and functions of these skills in NCAA Division I (DI) individual and team sport athletes.

More specifically, the following research questions were examined:

(1) Are there differences in the amount of time spent in relaxation and performance imagery skills by sport type?
(2) Are there differences in the levels of deliberate practice (relevance, concentration, and enjoyment) with relaxation and performance imagery skills by sport type?
(3) Are there differences when relaxation and performance imagery skills are used by sport type?
(4) Are there differences in the functions of relaxation and performance imagery skills by sport type?

## 2. Materials and Methods

### 2.1. Participants

This study included a total of 156 collegiate NCAA DI athletes from across the United States; however, only 117 were included in the data analysis, as they completed at least one section of the survey. All participants were required to be currently attending a university/college in the United States, playing a DI sport, active on their sport roster, and over the age of 18 years old. Participants were excluded if they did not meet these criteria. This study included 29 males and 88 females. Of the group, 45 participants played individual sports, and 72 participants played team sports based on self-identification. The average age of participants was 20 years old *(M* = 20.04, *SD* = 1.44), with the range of ages between 18 and 24 years. White or Caucasian was the predominate race (*n* = 97) of the participants included in this study, followed by Black or African American (*n* = 10), American Indian or Alaskan Native (*n* = 2), Asian (*n* = 2), and other (*n* = 6). Of the participants, 24 responded they did work with the SPP, 67 did not, but their school has an SPP, 23 did not, and their school does not have an SPP, and three were not sure. 

### 2.2. Instrumentation

The instrument was developed for this study based on prior deliberate practice research [4,10]. This study’s method was descriptive in design. For this research, The Deliberate Relaxation for Sport Survey was modified to fit the scope of the study by removing certain questions that were not relevant. Additionally, the researchers added performance imagery under each subsection. This questionnaire included six subsections: (1) extent of engagement in relaxation and performance imagery activity type, (2) perceived relevance, enjoyment, and concentration associated with relaxation and performance imagery activities, (3) engagement in relaxation and performance imagery activities during and outside of practice and competition, (4) functions of relaxation and performance imagery activities, (5) how relaxation and performance imagery activities were learned, and (6) extent of engagement in relaxation and performance imagery activities over athletic career. 

For the extent of engagement, participants were asked to rate the approximate time in minutes spent in each relaxation activity per week on a scale from 0 to over 120 min per week, grouped into increments of 15 (i.e., 0, 1–15, 16–30, and 31–45 min). Deliberate practice included four levels: sport performance, competing effectively, enjoyability, and concentration. Participants were asked to answer on a scale of 0 (not at all) to 5 (moderately) to 10 (highly) the extent to which they thought their relaxation activities were relevant to different areas of deliberate practice (i.e., sport performance, competing effectively, enjoyable, and mental concentration). A higher score, such as 8 or 9, would mean that the athlete felt that relaxation and performance imagery activities were relevant to that level of deliberate practice. Participants were asked to rate the extent of their engagement in relaxation activities and performance imagery at different time points (i.e., practice, competition, and outside of practice and competition) on a scale from 0 (never) to 4 (always). For the functions of relaxation and performance imagery, participants were asked to rate the extent to which they engage in relaxation activities and performance imagery for different purposes (i.e., coping with anxiety at competitions, everyday psychological stress, physical recovery, to enhance mental skills, automate preperformance routines, for mental focus, performance strategies, to correct mistakes, and learn and practice sport skills) on a scale from 0 (never) to 4 (always). 

Exploratory questions were asked at the end of the questionnaire to investigate how athletes learned relaxation and performance imagery and the engagement of each over their athletic career. Participants were asked to rate on a scale of 0 (strongly disagree) to 4 (strongly agree) the extent to which they agreed with questions asking about the method that they learned relaxation activities and performance imagery. An option to select “N/A” was offered in case the method did not apply to them or they did not use relaxation activities. 

### 2.3. Procedure

Before data collection began, IRB approval was obtained. The researcher compiled a list of all DI athletic staff and coaches, including athletic trainers, support staff, strength and conditioning coaches, graduate assistants, and head coaches. The researcher emailed each staff member with information pertaining to the survey and asked if they would be willing to pass along the survey to their athletes. If they agreed, a second email was sent to be forwarded to the athletes, which contained a link to the survey. Each contact was emailed twice, once in the fall and once in the spring, in case they were in season during the first round of recruitment and not able to participate. Additionally, recruitment occurred through word of mouth by asking personal connections of athletes and coaches if they would be willing to participate.

The Qualtrics survey began with an informed consent form for all participants. After providing consent, the participant completed the demographic survey and the Deliberate Relaxation for Sport Survey. The completion of the questionnaire took between 10 and 15 min. After the participant completed the survey, their response was recorded through Qualtrics. The participants were debriefed and thanked for their participation. 

### 2.4. Data Analysis

SPSS Statistics software was used to analyze the data. Descriptive statistics were used to analyze the demographic information. The subsections of the questionnaire were classified as the dependent variables of the study, while sport type were classified as the independent variables. 

An independent sample t-test was used to investigate differences in the amount of time spent on the different relaxation and performance imagery skills between sport types. A multivariate analysis of variance (MANOVA) compared the differences between the levels of deliberate practice, engagement in relaxation and performance imagery, and the functions of relaxation and performance imagery by sport type. 

## 3. Results

### 3.1. Time Spent in Relaxation and Imagery

**Figure 1:**
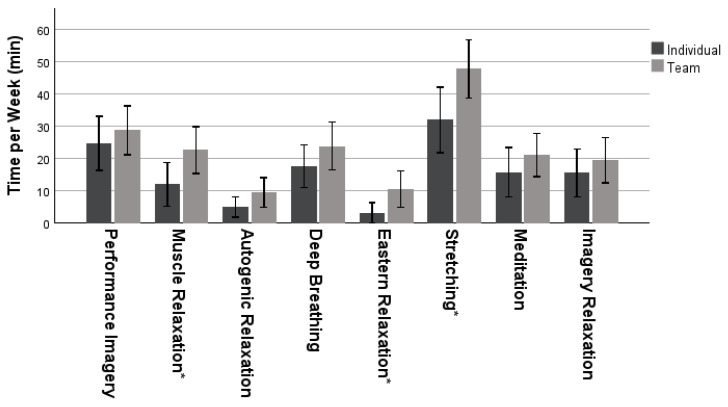
Mean ± 95% confidence intervals of time spent on relaxation activities and performance imagery by sport type. Note * = *p* < 0.05.

This section included 45 individual sport athletes and 71 team sport athletes (*n* = 116). An independent samples t-test investigated differences in the amount of time spent on the different relaxation skills between sport types, as seen in Figure 1. There was a significant difference in the time spent in stretching activities between individual sport athletes (*M* = 32.02, *SD* = 33.70) and team sport athletes (*M* = 48.51, *SD* = 38.62); *t* (102.910) = −2.433, *p* = 0.020. Team sport athletes (*M* = 22.69, *SD* = 30.63) used muscle relaxation activities far more each week than individual- sport athletes (*M* = 12.07, *SD* = 22.60); *t* (111.280) = −2.143, *p* = 0.034, equal variances not assumed. Again, team sport athletes (*M* = 11.81, *SD* = 26.01) typically used eastern relaxation activities more often than individual sport athletes (*M* = 3.07, *SD* = 11.13); *t* (104.253) = −0.507, *p* = 0.014, equal variances not assumed. 

### 3.2. Levels of Deliberate Practice of Relaxation and Performance Imagery

**Figure 2:**
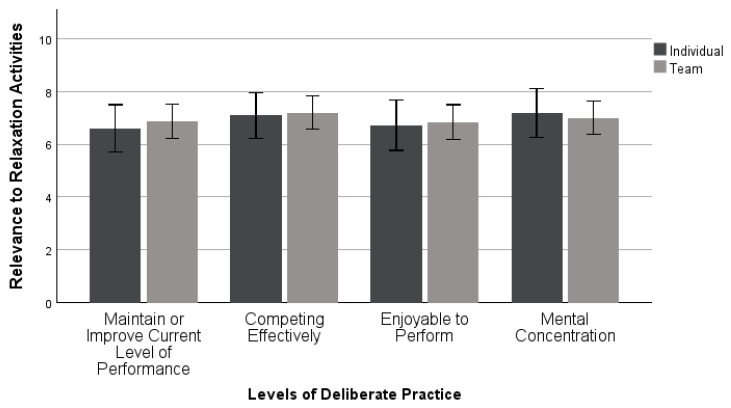
Mean ± 95% confidence intervals of deliberate practice in relaxation activities between sport types.

A MANOVA examined differences between the levels of deliberate practice of relaxation activities by sport type. This analysis included 43 individual and 66 team sport athletes (*n* =109). There was not a statistically significant difference found in levels of deliberate practice based upon an individual’s sport type: *Wilk’s λ* = 0.982, *F* (4, 104) = 0.198, *p* = 0.939, *η_p_*^2^ = 0.008. The results, as seen in Figure 2, indicated athletes found relaxation activities moderately relevant to each of these four aspects of deliberate practice. 

**Figure 3:**
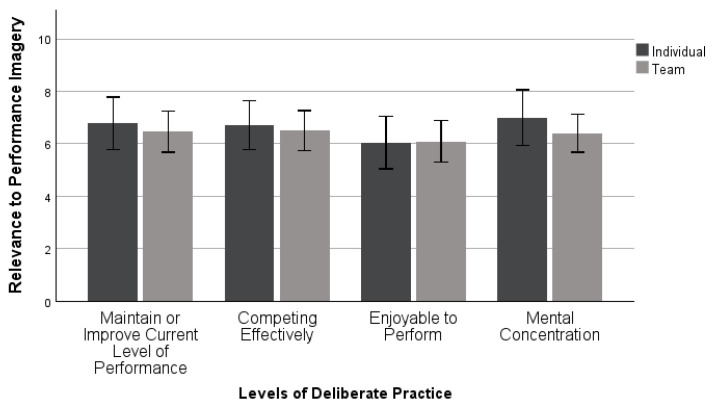
Mean ± 95% confidence intervals of deliberate practice in performance imagery between sport types.

Similarly, a MANOVA examined differences between the levels of deliberate practice of performance imagery by sport type. This analysis included 42 individual and 63 team sport athletes (*n* = 105). There was not a statistically significant difference found in levels of deliberate practice based upon an individual’s sport type: *Wilk’s λ* = 0.980, *F* (4, 100) = 0.512, *p* = 0.727, *η_p_*^2^ = 0.020. The results indicated athletes found performance imagery activities moderately relevant to each of these four aspects of deliberate practice, as seen in Figure 3.

### 3.3. Frequency of Engagement in Relaxation and Performance Imagery 

**Figure 4:**
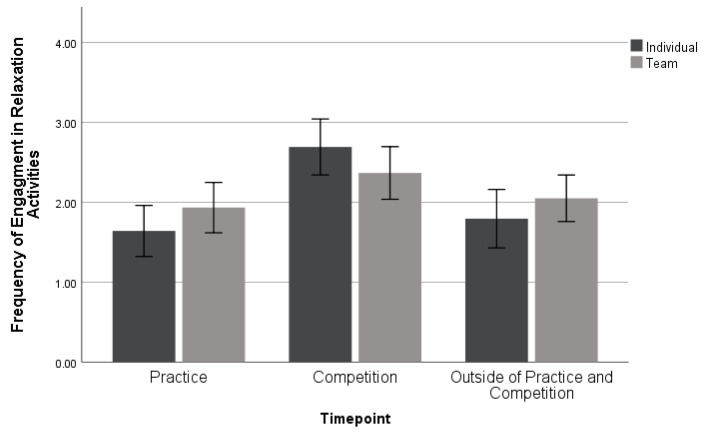
Mean ± 95% confidence intervals of differences in engagement of relaxation activities across sport types.

A MANOVA was used to examine the extent of their engagement in relaxation activities and performance imagery over different time points (i.e., practice, competition, and outside of practice and competition) by sport type. This analysis examined 39 individual and 60 team sport athletes (*n* = 99). No significant differences were found between individual and team sports when they used relaxation skills: *Wilk’s λ* = 0.927, *F* (3, 95) = 2.496, *p* = 0.064, *η_p_*^2^ = 0.073, as seen in Figure 4. Both team and individual sport athletes used relaxation most during competition.

**Figure 5:**
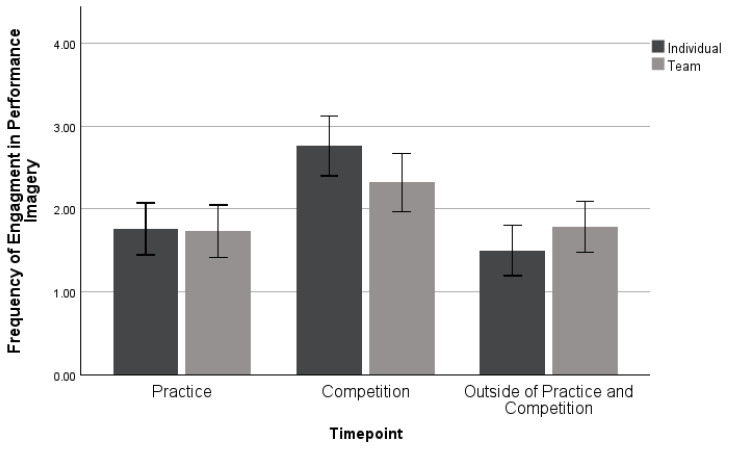
Mean ± 95% confidence intervals of differences in engagement of performance imagery across sport types.

A MANOVA was used to examine differences between sport types when they used performance imagery. Investigating engagement in performance imagery, this analysis included 42 individual and 56 team sport athletes (*n* = 98). No significant differences were found between sport types when performance imagery was being used, *Wilk’s λ* = 0.939, *F* (3, 94) = 2.036, *p* = 0.114, *η_p_*^2^ = 0.061, as seen in Figure 5. Overall, athletes use performance imagery most often during competition, with no differences between sport types. 

### 3.4. Functions of Relaxation and Performance Imagery 

**Figure 6:**
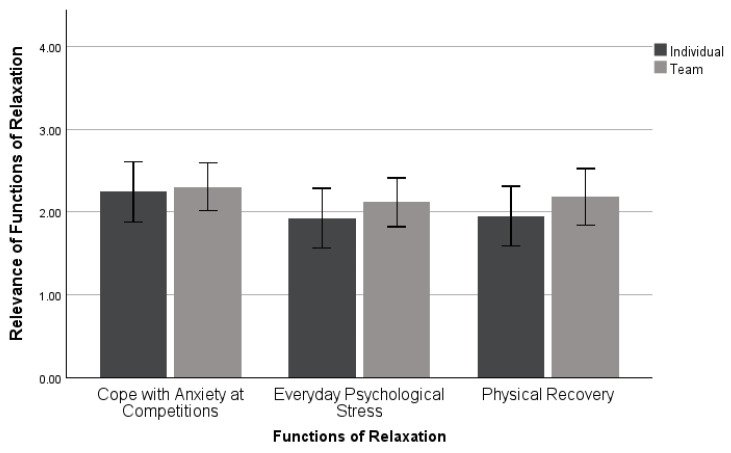
Mean ± 95% confidence intervals of differences in the functions of relaxation activities across sport types.

A MANOVA was used to investigate differences in the functions of relaxation activities by sport type. This analysis included 41 individual and 59 team sport athletes (*n* = 100). No significant differences were found in the differences in functions of relaxation skills by sport type: *Wilk’s λ* = 0.989, *F* (3, 96) = 0.358, *p* = 0.784, *η_p_*^2^ = 0.011, as seen in Figure 6. 

**Figure 7:**
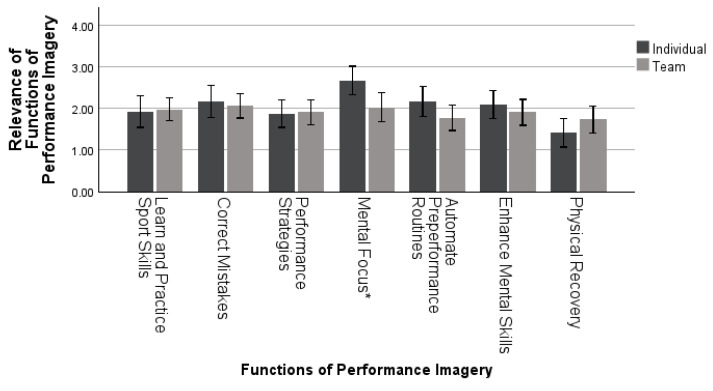
Mean ± 95% confidence intervals of differences in the functions of performance imagery across sport types. Note * = *p* < 0.05.

A MANOVA was used to examine differences in the functions of performance imagery by sport type. This analysis examined 41 individual and 58 team sport athletes (*n =* 99). MANOVA analysis revealed sport type had a significant effect on the function of performance imagery: *Wilk’s λ* = 0.811, *F* (7, 91) = 3.039, *p* = 0.006, *η_p_*^2^ = 0.189, as seen in Figure 7. Specifically, sport type had a significant effect on performance imagery when used for mental focus (*p* = 0.012). Individual sport *(M* = 2.68, *SD* = 1.08) athletes used performance imagery more often for mental focus than team sport athletes *(M* = 2.04, *SD* = 1.34).

### 3.5. Exploratory Questions

Exploratory questions investigated how athletes learned relaxation and performance imagery and the engagement of each over their athletic career. For how athletes learn relaxation activities, the results were scattered evenly across all three responses: taught the activities by a professional (*M* = 1.95, *SD* = 0.87), taught myself the activities (*M* = 1.56, *SD* = 0.86), and taught by trying things out (*M* = 1.82, *SD* = 0.90). These responses all varied from disagree to agree. The method participants agreed with the most was being taught performance imagery by a professional (*M* = 2.01, *SD* = 0.86). Teaching myself (*M* = 1.50, *SD* = 0.93) and learning by trying things out (*M* = 1.73, *SD* = 0.92) both fell, on average, between disagree and agree.

When asked how many years they have been using relaxation activities over their athletic careers, the average was approximately four years *(M* = 4.24, *SD* = 3.26). Similarly, participants, on average, have been using performance imagery for about four years (*M* = 4.24, *SD* = 2.92). This may indicate that individuals are learning these skills at the same time.

## 4. Discussion

The purpose of the current study was to better understand the relevance of relaxation and performance imagery skills to athletic performance as well as the extent of use, types, and functions of these skills in NCAA DI individual and team sport athletes. The present study investigated these differences, as well as how DI collegiate athletes are learning and using these skills in their sport. Previous studies have focused in a broad sense on how athletes use psychological skills in their performances [3,11,12,13,14]. However, there is limited research on how athletes use these psychological techniques in their sport, how they learn imagery and relaxation, and what these skills offer for their performances. Additionally, there is conflicting research on whether or not there is a difference in terms of sport type. 

Regardless of sport type, this study found that stretching was found to be the most popular relaxation activity among all athletes, which may be because this is something that every athlete knows and is an essential part of a proper warm-up and cool-down after a performance [20]. Because of the calm nature of stretching, this is an easy time for athletes to find some relaxation. Additionally, deep breathing is a technique that anyone can utilize and has immediate physiological impacts on heart rate [3]. Participants in this study used autogenic relaxation the least, which could be due to the more advanced nature of the activity. This type of relaxation is harder to learn on your own, requires a quieter space, and cannot be used during competition [21]. Athletes spend, on average, about 28 min per week in performance imagery, which shows they are using it somewhat regularly. The results from this research support the idea that DI collegiate athletes use relaxation and imagery skills in their sport. However, they may lean towards skills that can be used quickly and in a public space. 

Of these relaxation skills, team sport athletes use muscle relaxation, eastern relaxation, and stretching activities more often than individual sport athletes. Team sport athletes typically warm up and cool down together, which may allow them to perform these relaxation activities more frequently. Also, if one athlete on the team is doing a relaxation activity, it is likely the rest of the team will follow suit. In an individual sport, it is up to the athlete to decide on their own if they want to partake in that activity or not. However, more research is needed on this subject to fully understand why team sport athletes are using relaxation more often. Again, both sport types spend a similar amount of time in performance imagery, which could be due to the nature of collegiate sports and the high demands of any sport. This contradicts previous findings [15,16,17] stating that team sport athletes use imagery more frequently than those in individual sports.

Both individual and team sport athletes are using relaxation in a deliberate way, rating all functions moderately relevant to their practice. Additionally, deliberate practice is enjoyable, which is in agreement with some previous research [4,19]. However, this contradicts the original deliberate practice framework from Ericsson and colleagues [18]. This framework is from 1993, which could mean that over the past 20 years, the way that individuals are practicing skills has changed, specifically that they are able to fully concentrate but also find it enjoyable. Because the scores from these participants are not on the higher end, this could indicate that they are not always deliberately using relaxation and performance imagery for these four purposes. Across sport types, relaxation and performance imagery were used in similar manners, demonstrating a moderate use of deliberate practice, meaning that athletes may not be fully invested and concentrating when using these skills with purpose. It is important for athletes to use relaxation techniques and imagery in a deliberate manner so that they may obtain the benefits for their performances and increase their self-awareness [4]. 

This study found that athletes most commonly use relaxation and performance imagery during competition, regardless of sport type. This is something seen commonly among other research studies, as athletes only deploy psychological skills during high-stress scenarios [9]. From previous research [4], it is known that the more athletes use psychological skills, the more benefits are seen, such as developing mental toughness. Just like physical skills, both imagery and relaxation skills require consistent practice to truly master and gain benefits from. The more that athletes use psychological skills prior to competition, the more likely they are to see themselves as successful [8]. SPPs should encourage athletes to use these techniques before and during practice as well as outside of practice and competition. 

Sport type had a significant impact on the function of performance imagery, specifically for mental focus. Individual sport athletes tended to use performance imagery more often for mental focus compared to team sport athletes. While previous research with sport type has investigated frequency [15,16] and capabilities of performance imagery [2], there is still more research needed examining the functions of performance imagery. One explanation of this finding is that individual sports tend to require much more concentration for longer periods of time due to the absence of team dynamics with group sports such as soccer or football. With sports like golf or swimming, the individual may require more mental focus on one’s abilities due to fewer interruptions of the sport.

This study aimed to explore how athletes learn relaxation and performance imagery activities, as well as their engagement in those activities over their athletic careers. An even mix of answers was given for how athletes learned relaxation activities between being taught by a professional, teaching themselves, and being taught by trial and error. This demonstrates athletes are learning relaxation techniques through a variety of ways. This may be dependent on the skill they are learning, as deep breathing and stretching are very intuitive or basic, while autogenic or progressive muscular relaxation is more advanced. However, for performance imagery, most athletes learned this skill from a professional. Performance imagery is a complex task to learn, and many people have not heard of it until they work with someone in the field of sport psychology. The SPP can help the athlete to develop the proper techniques along with how and when to use this skill as well, whereas, without their help, athletes may struggle with knowing how to use imagery for performance benefits. 

On average, athletes had been participating in relaxation and performance imagery for four years, indicating that many of them had learned these skills at the same time. Some types of imagery can be used as relaxation techniques [22], so many athletes may have learned this skill as a result of learning relaxation skills. The natural progression would be to learn more types of imagery, such as performance, during this time as well. While some techniques are easier to learn by oneself than others, it is important for SPPs to teach athletes these skills in order to help athletes understand the proper uses, the correct techniques, and different strategies for when to use each technique in their sport. Athletes may be teaching themselves these skills and not actually getting any benefits from them because of improper use. Out of the 117 participants, only 24 responded they work with the SPP; 67 did not work with one, but their school has one, and 23 said that they did not work with one and their school does not have an SPP. It is interesting to note that most athletes in this study do not work with the SPP, though they have access to one. Previous research found that collegiate student athletes’ openness to mental skills training and support from SPPs was dependent on sex, sport type, and previous experiences [23]. In general, females are more receptive than males, and athletes who had highly effective prior experiences were more open to working with an SPP on mental skills. Team sport athletes are interested in mental training focusing on improved communication and building team cohesion, while individual sport athletes are more open to mental training that helps them perform as well in competition as in practice. The results from the current study could be due to the continued stigma of mental health problems that many associate with seeing an SPP. If the school has an SPP, it is important for the coaches to encourage utilization of their services.

## 5. Limitations and Future Research

Several limitations to the present study are worth mentioning. First, all measures were self-reports; therefore, the data that were collected only reflect the perceptions of the athletes taking the survey. These data may not be entirely accurate, as participants may respond to the survey according to what they think is socially desirable or what their coach may think. The study could be improved by incorporating journals and observations from the participants of the studies that would allow researchers to collect more in-depth data concerning their habits and uses of relaxation and performance imagery strategies across multiple time points. Additionally, incorporating interviews with coaches, SPPs, or athletic trainers could provide more information about the athletes’ activities. Because this questionnaire was answered anonymously and was sent to university contacts twice, there is a chance that participants may have completed the questionnaire more than once. While participants were asked to only complete the questionnaire once, this is a limitation to the data. 

While it was the goal of the study to balance sex and sport type groups evenly, more females were included than males, and there were more team sport athletes than individual sport athletes, therefore not making this a representative sample. Future research should balance sex as well as sport type. Some caution should be used when interpreting the results of this study because the time of the season during which data were collected varied. Some teams had just finished their season, while others were still in season. Moreover, the various collegiate divisions were not included. Also, participants may have responded a certain way since the survey came from their coach despite being told that their answers were anonymous. Future research should address these limitations. Additionally, another area of research should focus on why athletes are not utilizing the SPP if their school has one and, possibly, how to better integrate the SPP into the athletic system.

## 6. Conclusions

The present study provided knowledge into how DI collegiate athletes use psychological skills to cope with the demands of their sport and how similar individual and team sport athletes are in the way they use these relaxation and imagery skills. Research continues to support the benefits of relaxation and performance imagery in sports as ways to enhance performance [24,25]. We see that changes have occurred over the last 20+ years of literature, and new research needs to be reviewed to learn more about how current athletes are using these skills. Relaxation and performance imagery are both powerful skills that can reduce anxiety, increase peak performance opportunities, achieve optimal focus, and allow the athlete to enjoy their sport and performance once again. 

Collegiate athletics, especially at the DI level, is incredibly stressful due to the amount of attention the athlete receives, as well as being treated like professional sports with the added responsibilities of being a full-time student. The current research provided evidence that individual and team sport athletes are more similar than different in the way that they use relaxation and performance imagery activities. However, understanding that team sport athletes are spending more time in stretching, muscle relaxation, and eastern relaxation activities could be beneficial when implementing psychological skills with a DI sports team. Both individual and team sport athletes are using relaxation skills and performance imagery deliberately and intentionally, as well as finding them enjoyable. Also, since both types of athletes commonly use these skills only during competition, the SPP can stress the importance of practicing and utilizing these skills during practice and downtime as well. Lastly, understanding that individual sport athletes use performance imagery more for mental focus purposes could help direct how the SPP teaches or explains imagery skills to those athletes. Overall, psychological skills such as relaxation and performance imagery can assist DI student athletes to thrive in their sport and college experience when implemented correctly.

## Data Availability

The data presented in this study are available on request from the corresponding author.
